# Impact of Mobile Games-Aided Neurorehabilitation: A Systematic Literature Review

**DOI:** 10.21315/mjms2023.30.6.4

**Published:** 2023-12-19

**Authors:** Ruvenaa Sagary, Nurul Hashimah Ahamed Hassain Malim, Nasuha Lee Abdullah, Wan Nor Azlen Wan Mohamad, Alwani Liyana Ahmad

**Affiliations:** 1 School of Computer Sciences, Universiti Sains Malaysia, Pulau Pinang, Malaysia; 2 Department of Neurosciences, School of Medical Sciences, Universiti Sains Malaysia, Kelantan, Malaysia; 3 Hospital Universiti Sains Malaysia, Kelantan, Malaysia

**Keywords:** cognitive skills, mobile games, motor functionality, neurological rehabilitation

## Abstract

Neurological rehabilitation is a physician-supervised programme for individuals with nervous system diseases, injuries or disorders. Neurological rehabilitation, also known as neurorehabilitation, is part of the rehabilitation process that improves function, reduces severity and enhances a patient’s well-being. Because neurological injuries occur in the brain, spine and nerves, affecting multiple body parts including organs, blood vessels, muscles and bones, rehabilitation requires a multidisciplinary approach. This study conducted a systematic literature review (SLR) on the use of mobile game in neurorehabilitation. The steps undertaken in the literature review included the collection, identification, categorisation, summarisation and synthesis of relevant studies in the research domain. A total of 50 related articles were reviewed. The study identified that the effects on cognitive skills, handgrip strength, memory, attention, visuospatial abilities, executive function tasks, motor functionality, and improvements in balance, visual perception, and functional mobility are impacts of the use of mobile games in neurological rehabilitation. Furthermore, several research challenges and recommendations for future research were identified.

## Introduction

Neurological injuries include injuries to the cerebrum, spine or associated nerves. More than 600 types of neurological disorders exist, such as traumatic brain injury, which causes headaches, stroke or seizures, multiple sclerosis, Parkinson’s disease and cerebral palsy. Upper limb impairment in the chronic stage affects over 75% of patients with stroke (1). These impairments can be hereditary, passed down from parent to child through genes or present at birth. A neurological injury can result in any degree of impairment. The incidence rate of facial palsy, which affects individuals of all ages and sexes (range: 11.5–53.3 per 100,000 people), depending on the population (2–4). Brain and spine injuries can occur due to external sources such as hard forces on the head that cause head injuries. These types of injuries may cause side effects, such as obscured vision or loss of consciousness. In extreme cases, injuries can push affected individuals into a vegetative state. The specific causes of neurological injury vary and can be caused by genetic defects, congenital anomalies or disorders, diseases, lifestyle or environmental health problems, malnutrition, brain damage, spinal cord injury, nerve injury or reactions to gluten. Brain damage caused by stroke frequently affects the parietal, frontal, midbrain, and brainstem regions, resulting in memory, language, executive, and attention dysfunctions. This dysfunction leads to cognitive impairment and motor deficiencies that substantially influence day-to-day activities (5). The signs and symptoms of neurological injuries differ according to the severity of the injury and the affected body parts. The causes of neurological injuries may also differ. Although brain and spine injuries, commonly caused by trauma or accidents, can cause neurological disorders, other neurological disorders may also be caused by genetic diseases, infections, or lifestyle-related factors.

A genuine handicap in a patient caused by serious brain or spinal injuries unexpectedly disrupts their family’s capacities, way of life and ventures. The patient and their family started a new style of living in their larger local area with their transformed bodies, and as a different person to adapt to the present situation. Recently, there has been an increase in the adoption of novel rehabilitation strategies to enhance arm and hand functionality in patients with multiple sclerosis (6–8). Playing is one of the most effective ways for people to learn. Moreover, playing is the preferred method of education among young children and continues to be a fulfilling and pleasurable activity for adults (9). Neurorehabilitation deals with the skills and mindset of individuals with disabilities and their loved ones. Neurorehabilitation enhances an individual’s ability to act with an essential degree of autonomy possible for them. Moreover, it helps restore confidence and develop a healthy mindset. They will adapt to new circumstances and engage in fruitful and submitted reintegration in the local region. Another study revealed that corticomotor excitability was positively related to an hour of tongue-task training (10). A similar finding has been demonstrated in several animal studies on motor-skill training (11, 12). Therefore, rehabilitation training may result in changes in cortical structure after facial palsy.

To provide the most balanced consideration for patients, numerous neurorehabilitation treatments, regardless of whether offered by clinics or private healthcare facilities, should be considered by numerous specialists in various fields. Throughout a certain period and occasionally over an individual’s lifetime, these medicines conceivably permit the individual and the group of that individual to live the most typical, autonomous life. For instance, there is a great deal of interest in using technology to overcome the difficulties associated with dementia. Despite the abundance of technology, from full-fledged smart home assessment settings to companion robots, the development and implementation have been gradual or inconsistent (13).

Interactive computer games featuring components of both physical and mental training have been extremely motivational for encouraging children to engage in physical exercise at a given level for extended periods. Advancements in neuroimaging techniques have substantially improved the degree and outcome of neurorehabilitation. Information and communication technologies (ICT) are extremely important for assisting in the rehabilitation of individuals with disabilities (14). Telerehabilitation (TR) is the provision of rehabilitation services utilising electronic systems and ICT in the context of eHealth (15). Besides, TR provides rehabilitative care outside of the hospital context in an eco-friendly environment, assisting in identifying new constraints and assessing the success of the intervention concerning the activities of daily living (ADLs) at sustainable costs.

Researchers are currently using neurorehabilitation innovations to provide first-line changes to the care of patients with sensory system disorders. In particular, the use of mechanical technology in neurorehabilitation is becoming increasingly common. Approximately 75% of patients with brain injury aged ≤ 35 years old and have adequate computer and mobile device competence (16). Re-enactments in computer-generated simulations and computer games offer patients a chance to vividly experience and relearn different aspects of their lives and conditions while being watched by their clinicians and specialists. In addition to other automated advances, these devices and reproductions provide opportunities for preparation. Exercise-based recuperation would be faster for patients who have recently experienced stroke or other cerebral or spinal string injuries, thereby shortening the recuperation time. Technology acceptance describes how users perceive, accept and embrace technology because it is intended to assist (17). Recently, virtual reality (VR) has become a potentially useful tool in several therapies and rehabilitation domains (18, 19).

According to scientific studies, playing video games may modify a person’s level of enjoyment, attentiveness and dominance, which changes their perception of well-being (20–22). In addition, video games that are easy and simple to play are more readily accepted and produce pleasant feelings in older individuals (23). Serious games and VR are viable rehabilitation techniques for improving upper limb function in individuals with neurological illnesses. Crucially, they adapt well to home-based rehabilitation (24).

## Methods

Various studies have reported on the use of mobile games for neurorehabilitation. Hence, this study attempted to analyse the effects of using mobile and video games in neurorehabilitation. This systematic literature review (SLR) followed the guidelines of Shute et al. (25), which define SLRs as studies to plan, review and analyse relevant information or data on a particular topic, query or research area. This study aimed to identify the positive and negative outcomes of the existing technological devices for neurorehabilitation. The suggested methods are as follows:

- Determine the research question
- Identify relevant studies or articles
- Extraction of data on relevant platforms (ScienceDirect, IEEE)
- Note relevant information
- Study selection
- Analyse the article or study and summarise the results
- Discussion that includes the conclusion

### Eligibility Criteria

The SLR, which drives the entire process, must be guided by a research question. The methodology and the extracted and derived data must be capable of answering the research questions. Several researchers have recommended developing a specific research question and selecting a subtopic for the SLR (26). Research questions should not be too specific because there may be few articles and an SLR cannot be performed with a small sample.

The SLR aims to understand the current knowledge domain of using mobile games for neurorehabilitation. Table 1 lists the five research questions (RQ) that have been identified to guide this study. The outcomes of the SLR are expected to help determine future research directions and highlight challenges in the field.

### Search Strategy

The SLR was performed through an electronic search using the PubMed and Google Scholar databases. All datasets were searched from the foundation of the data set to 25 January 2021. The primary terms include ‘neuroinjuries’, ‘neurorehabilitation’, ‘computer games’, ‘VR’, ‘virtual reality’, ‘Wii’, ‘Kinect’ and ‘Xbox’, and their connected equivalents were searched.

To identify articles, the initial step was to search for articles in the datasets and examine the titles and abstracts. The subsequent step was the exclusion of studies utilising the title or theoretical and inclusion criteria. The final step was to summarise the full content of the qualified articles.

### Selection Process

Articles published in English that met the following requirements were selected:

- study of the technical devices used (VR, Kinect and Wii)
- discussion on the assessment of neurorehabilitation therapy
- disease history is linked to neurological injuries
- software or hardware games
- the game used in the study serves neurorehabilitation purposes

No limitations in sample size were made. Articles that failed to meet the following criteria were excluded:

- did not have a database (books or theoretical papers)
- not published in English
- articles that did not directly define neurorehabilitation
- technical devices have not been emphasised as clinical intervention devices

Statistical data from quantitative studies were also pooled within the meta-analyses. In addition, articles involving technological devices for assessment or recovery, neurological diseases, cognitive rehabilitation and studies surveying neuropsychological assessments before and after mediation were included.

### Data Abstraction

The entire text of the selected studies was examined to determine whether it fulfilled the exclusion and inclusion criteria. Data were independently extracted after identifying relevant articles for inclusion in the analysis. The extracted contents included: i) article title, ii) author’s name, iii) year of publication, iv) exclusion and inclusion criteria, (v) research objects, (vi) intervention measures, (vii) control measures and (viii) results.

## Results

The search strategy generated 52 references from two electronic databases based on titles and abstracts. The articles were deemed relevant, and the complete text of each study was analysed. A total of 15 articles were included in the meta-analyses and reviews based on the articles. Nevertheless, 37 articles were based on experiments conducted by researchers.

The main outcomes of the articles were positive, as they supported the idea of using technological devices, video games and VR-based games to treat neuroinjuries during neurorehabilitation. Five articles had inconclusive conclusions owing to the need for further research on this topic. Table 2 summarises the characteristics of the selected articles.

### Cognitive Skills

Regardless of physical function deficiencies, cognitive difficulties manifest as problems with coordination, attention, dual-tasking, processing speed, and visuospatial and working memory conceptualisation (23). Cognitive skills were one of the main outcome measures. Executive functioning, organisation, attention, problem-solving, memory and visual processing are a few cognitive domains (27). Attention is a fundamental component of cognitive function (28).

Cognitive functioning is related to an individual’s capacity to process their thoughts. Memory, speech and the ability to pick up new information are all areas in which cognition is relevant. The brain is normally capable of acquiring new abilities and creating personal thoughts and beliefs about the world in the areas mentioned earlier, often during early childhood. Age and illness can affect cognitive functioning, leading to memory loss and trouble choosing the right words to use while writing or speaking. For instance, multiple sclerosis may eventually result in memory loss, reduced capacity to comprehend new ideas or information and a lack of verbal fluency. Based on this analysis, 15 articles measured the impact of cognitive skills from a neurorehabilitative perspective. These studies concluded that rehabilitation using video games and VR-based games positively affected cognitive functioning. Therefore, neurorehabilitation using video games is beneficial for enhancing an individual’s cognitive skills.

### Hand Grip Strength

Patients’ handgrip strength, hand function tests and upper extremity functions were also analysed. Research findings from articles measuring these criteria indicate that they are important in rehabilitation. Nevertheless, further research is needed to reach a definitive conclusion, as only a few such studies are available. These criteria, if included, would benefit the rehabilitation programme.

### Memory, Attention, Visuospatial Abilities and Executive Functions

Attention functioning, also referred to as executive functioning, is another subtopic with similarly high outcomes. Attentive control refers to an individual’s ability to decide what they will give attention to or disregard. This is known as endogenous or executive attention. A set of mental abilities known as executive functions includes working memory, flexible thinking and self-control. Human use these abilities to learn, work and govern their daily lives. Focusing, following instructions and managing emotions may be among the many things that people with executive function issues find challenging. The analysis concluded that attentional and executive functioning are important for an individual to pay attention, begin activities, concentrate until they are completed and engage in other behaviours.

Stroke is a medical emergency caused by neuroinjuries that requires treatment through rehabilitation. A stroke is characterised by ‘rapidly developing clinical symptoms and/or signs of focal and at times global loss of cerebral function, with symptoms lasting more than 24 h and no apparent cause other than that of vascular origin’ (29). Gait rehabilitation is a common type of rehabilitation for patients with stroke. Gait is the human walking pattern. Walking requires balance and muscular coordination to push the body forward in a rhythm known as a step. Gait training increases cardiovascular fitness, impedes adaptive changes in soft tissues in the lower limbs, and increases muscle strength and coordination. The analysis revealed that approaches to gait rehabilitation after stroke include motor learning and neurophysiological techniques, robotic devices and the newly evolving utilisation of the brain-computer interface, where positive outcomes and objectives are achieved during rehabilitation.

Visuospatial ability was another outcome of this analysis. Four of the reviewed articles analysed and validated its capability to be incorporated into neurorehabilitation to achieve good outcomes. Visuospatial ability is critical for functional movement that exercises learning is achieved through repetitive execution of the exercise. During this time, continual feedback and opportunities to fix or minimise faults are offered (30, 31). Visuospatial capability refers to the ability of a person to determine visual and spatial relationships between objects. The ability to visualise objects, construct larger shapes by identifying smaller components, and recognise distinctions and similarities between objects are examples of visuospatial abilities. One study used VR-based technologies to test a patient’s visuospatial abilities and concluded that they were beneficial for memory rehabilitation. Therefore, therapists can use video games for the rehabilitation of patients to test their visuospatial abilities, and determine and improve their capabilities.

### Motor Functionality

The second most frequently measured outcome was the impact on motor functioning. Motor functioning is an essential criterion for therapists to test patients before therapy initiation. The abilities to learn or demonstrate skilful and successful assumptions, maintenance, adjustments and regulation of voluntary postures and patterns of movement are motor functions. Physical therapists use tests and assessments to determine fatigue, paralysis, patterns and postures of dysfunctional motion, erratic pacing, poor balance, clumsiness, and the capacity of individuals to regulate voluntary postures and movement patterns. Monitored responses at rest, during and after an activity can indicate the existence or intensity of a disability, limitation of activity or restriction of participation. Repeatedly performing motor activities that are important to the patient encourages functional recovery and fosters the autonomous realisation of trained skills (32, 33).

Based on this analysis, articles that measured motor functioning outcomes proposed that therapy based on VR, video games and software aimed at neurorehabilitation increases the efficiency of motor functioning in individuals, that is supported by findings from various studies. According to the literature, when patients ‘focus’ on the game rather than on their incapability, exercises become more pleasurable, encouraging and motivating to retain their performance in numerous trials and for an extended rehabilitation period, which is required to stimulate plastic changes in the central nervous system (34).

Therefore, the entertaining and engaging design of VR games may have encouraged increased participation from patients (35, 36). Gaming programmes make rehabilitation therapy enjoyable and interesting and help advance motor learning (30). By utilising the player’s natural feeling of competitiveness and need for engagement, games also stimulate, encourage and inspire interest in rehabilitation while encouraging learning processes. Games offer interactive stimuli and feedback that are essential for acquiring motor skills (37). Furthermore, individuals with psychiatric disorders, traumatic brain injuries and stroke benefit more from rehabilitation when they are highly motivated (38).

### Balance, Visual Perception and Functional Mobility

Balance is the ability to retain the mass centre point of the body over its support base. A well-functioning balancing system allows humans to see clearly while moving, define gravity orientation, assess motion direction and speed, and perform automatic postural changes in different environments and activities to maintain posture and stability. To attain and maintain balance, a series of complex sensorimotor control systems work together to integrate sensory inputs from the vestibular system (motion, balance and spatial orientation), vision (sight), proprioception (touch) and motor outputs to the muscles of the eyes and body. One or more of these components may be affected by accidents, illnesses, medication use, or ageing. In addition to the effect of sensory information on the human feeling of balance, psychological factors may also play a role. Motor functioning is the third subtopic with similar outcomes and should be incorporated into neurorehabilitation.

Finally, the outcomes from the findings of the articles with the least conclusive similarity were processing speed and working memory. Working memory and processing speed are associated with cognitive skills, or motor and attentional functions. Therefore, the criteria have already been met and proved efficient in individuals with neurological injuries, even with results and efficiency.

## Discussion

Various devices and approaches have been used by researchers to study neurorehabilitation. These studies provide evidence that neurorehabilitation benefits from various technological aspects. The technological advances used in the articles were VR, Xbox, video games, iPad software, Parrot application, Lumosity application, mobile games, Wii Fit, Kinect, and physically played games. New technologies, such as VR, may enable the provision of programmes with easier activities for children to undertake independently.

When evaluating the selected studies, the findings revealed that multiple studies have proven the benefits of VR in therapeutic treatments. The findings revealed that 16 of 52 articles supported the statement that VR is an efficient option for neurorehabilitation. For instance, VR has shown positive outcomes in patients with spinal cord injury, Parkinson’s disease, stroke and brain injury. Recovery services are increasingly using technology, such as VR environments, to mimic real events and social experiences. The primary outcome of all the studies undertaken on the impact of VR on the rehabilitation of individuals with neurological injuries showed that VR benefits patients in terms of visuospatial abilities and executive functions in neurodegenerative and acute conditions. These physical and cognitive limitations typically necessitate multifaceted treatments involving various health specialists. Extensive and long-term training is required to achieve neuroplastic changes in the brain (39).

Several studies have commonly referred to VR as a computer-based technology offering visual feedback on a display. Nevertheless, VR is a high-end user-computer interface inclusive of real-time stimulation and an embedded participant’s experiences through various sensory channels (visual and auditory, often haptic, smell and taste, if possible), depending on the synthetic environment in which participants feel their senses.

The feasibility of particular consoles for stroke rehabilitation, such as the Nintendo Wii, Nintendo Wii Fit and Sony PlayStation 2 Eyetoy, has been studied and is capable of enhancing movements in patients with stroke (40, 41). Xbox is another form of rehabilitation device with proven productivity. Xbox includes video games that require hand and finger movements. After a 3-month video game intervention, physical activity markers improved in 24 patients with chronic stroke (42). Self-training using video games can enhance upper extremity function in patients with chronic and subacute conditions (43, 44). Based on this review, 15 articles have studied the efficiency of video games. Three other studies explored the use of the Xbox. Video game rehabilitation uses standard gaming consoles to target and address physical and mental limitations through corrective cycles. Video games are becoming increasingly integral to word-related treatments in intensive recovery and community settings.

The gaming console’s capability to exist everywhere and be purchased by any individual enables personalised therapy for the patient. Patients using gaming to expedite recovery tend to be more interested in counselling efforts and more likely than those participating in daily treatment efforts to resume therapy beyond the doctor’s office. These positive effects are produced by enabling them to receive therapy at the convenience of their own homes at a relatively reasonable cost. In addition, patients participating in gaming recovery have reported improved results owing to their increased commitment to treatment and willingness to continue therapy.

Kinect is another device that provides creative and fun ways to rehabilitate, recover rewards, and improve motivation and eventual adherence. According to the review, a study that used Microsoft™ Kinect^®^ V2 (Kinect) for their experiment concluded that Kinect is ideal for detecting angles of spine motion with no considerable pause in-game execution, providing data and reflecting the campaign the player performs faithfully. As technological advancements in the health sector continue to flourish and have become a part of the treatment options for therapy, physiotherapists are required to improve their expertise in this field. Furthermore, it decreases the workload by efficiently utilising physiotherapy time while still providing treatment.

Moreover, Nintendo Wii Fit is a suitable device for neurorehabilitation. According to the present review, Nintendo Wii Fit has proven to improve static and performance-related activities. Good balance is usually obtained using Nintendo Wii Fit balance games as an intervention method, thereby promoting their use in neurorehabilitative training. As a biobehavioural assessment and training system for balance capacity, Nintendo Wii Fit has attracted particular interest in the field of neurorehabilitation.

iPad-based software has shown promising improvements in individuals with neurological injuries. A touchscreen that responds to finger movements controls the iPad. The iPad also has several other functions that enable it to calculate and respond to movements. Based on the two studies reviewed, iPads affect individuals’ rehabilitation, especially for those with various impairments. Furthermore, no support was required to set up the iPad. The user can access the device and start playing immediately, providing a degree of dignity and ownership for the patient during recovery.

Two additional applications created solely for rehabilitating patients with neurological injuries were also reviewed. The Parrot Software comprises 75 distinctive programming programmes for memory deficits and the remediation of speech, attention, cognition, and language in people with aphasia stroke or cerebral injury. The Lumosity application was the second application reviewed. Lumosity is an application designed to keep the brain active and assist in healing by providing minimal mental exercise every day. Based on these articles, the efficiency of both applications has been verified.

## Conclusion

In conclusion, incorporating technology-based devices and games for the rehabilitation of individuals with neurological injuries is a good idea. Nevertheless, therapy and rehabilitation sessions require further research to identify the type of rehabilitation that should be performed during treatment. Not all gaming methods are suitable for all patients. Various methods and tasks have been developed to target specific body parts. The incorporation of technology-based devices and games will provide more advantages in terms of efficiency. The findings of this overview will provide clinicians, researchers, and therapists with guidance in identifying evidence-based outcomes.

Conventional rehabilitation also falls short of providing the degree of therapy required to fulfil a patient’s rehabilitation requirements (45). Brain injury is becoming increasingly common, especially in patients who have experienced major accidents (46). Although the present findings support the hypothesis that video games tend to be a more efficient rehabilitation method for patients with neurological injuries, multicentre randomised controlled studies with more meticulously planned, systematic interventions and increased sample sizes are necessary to establish a solid evidence-based foundation and evaluate the potential advantages of this neurorehabilitation method. The brain-computer interface can also be combined with robotic training for a top-down approach (6).

### Limitations

Although a thorough literature search was performed, this review had certain limitations. First, our review did not include articles in minority languages and grey literature. Second, the VR intervention forms, preparation, time and dosage, strength of training and indices of outcome assessment employed in the reviewed studies were distinct. Therefore, some variability was present in the data collection, which is a common constraint in other formal VR intervention assessments.

In addition, the games used in certain studies to conduct the observations differed. They did not cater to all patients with different impairments because the games did not include a variety of exercises. The outcomes of all the studies cannot be generalised, as most studies were not conducted with large sample sizes with patients from diverse backgrounds. Most observations were conducted in a rehabilitation centre, where people mostly belong to the same ethnicity. Furthermore, long-term results could not be obtained because of the time required to complete long-term research.

## Figures and Tables

**Table 1:** Research questions and motivations

RQ	Research question	Motivation
RQ 1	What are the effects of mobile games or video games aided neurorehabilitation on the cognitive skills of a neurologically injured patient?	To highlight positive and negative (if any) effects on the patient’s cognitive skills.
RQ 2	Is there a difference in handgrip strength tests before and after a patient undergoes mobile or video games aided neurorehabilitation?	To identify any improvements in the patient’s handgrip strength.
RQ 3	Is there a difference in executive functions, visuospatial abilities, memory, and attention tasks before and after a patient undergoes mobile or video games aided neurorehabilitation?	To highlight the impacts on patients’ executive functions tasks, visuospatial abilities, memory and attention.
RQ 4	Does mobile gaming or video game neurorehabilitation improve the motor function of a neurologically injured patient?	To identify any changes in the patient’s motor functionality after undergoing neurorehabilitation.
RQ 5	Are there any improvements in visual perception, functional mobility, and balance after the patient completes mobile gaming or video games aided neurorehabilitation?	To highlight the improvements in patient’s visual perception, functional mobility, and balance.

**Table 2:** Summary of characteristics of included articles

RQ	Year (Ref)	Pathology involved (rehab focus)	Devices (name)	Outcome
RQ 1	2013 (11, 12)	Memory impairment (Cognitive skills)	Cat versus Mouse (mobile game) Modified Trail Making Test (mobile game)	Participants’ mental attentiveness and well-being increased throughout the course of the three-month gaming session.
RQ 1	2015 (25)	Cognitive Non-cognitive skills	Portal 2 Lumosity	The ‘brain power index’ (BPI), the sum of a player’s scores across all Lumosity activities and constructs, served as the performance indicator in Lumosity. Moreover, includes constituent scores for speed, memory, attention, flexibility and problem-solving.
RQ 1	2014 (26)	Impairment-based	iPad-based software	All participants showed overall progression in their iPad-based therapy activities in terms of accuracy and task latency.
RQ 1	2017 (27)	Dementia	Software based assistive technologies	The method of systematic mapping is used for the study of the empirical data. The findings of the systematic mapping indicate that the SWAT offers a range of services to those with dementia, including cognitive support, reminders, leisure activities and health monitoring.
RQ 1	2013 (28)	Cognitive improvement	Online brain games	Provided inconclusive evidence that brain games improve functioning.
RQ 1	2016 (29)	Stroke (Cognitive functioning)	Computer-based brain training programme	Overall, this study’s findings and related studies suggest that CBCR therapies addressing one cognitive domain are highly effective to those targeting many cognitive domains in terms of far and near transfer effects for patients with stroke. Computer tasks must be strongly associated with the impaired task to improve their everyday activities.
RQ 1	2017 (33)	Cerebral palsy	Nintendo Wii	The intervention group showed a substantial improvement in upper limb functions, whereas the control group failed to display any improvement. No significant differences in terms of improvements in functional mobility, balance, and visual perception were observed between the control and intervention groups.
RQ 1	2019 (42)	Alzheimer disease (Cognitive function)	Digital biomarker technologies	Evidence from implanted passive sensors is the most advanced study topic, implying that some of these solutions may be presented to larger populations in the future decade. Increased focus on these technologies in the clinical and scientific sectors may be beneficial in future.
RQ 1	2019 (44)	Amnestic mild cognitive impairment (aMCI)	Game-based neurofeedback training (NFT) system	Conclusively, NFT therapy enhances sustained attention and SWM. Nonetheless, NFT showed no influence on pattern recognition memory or short-term visual memory, both of which are characteristics of aMCI. The NFT system used in this study may selectively enhance sustained attention, strategy, and executive skills, but not other cognitive impairments common in women with aMCI.
RQ 1	2018 (43)	Mild cognitive impairment	Technology-based cognitive training	Although technology-based rehabilitation and cognitive training approaches are promising, owing to the differences in research design the findings were contradictory.
RQ 2	2020 (21)	Spine	Xbox 360 Kinect^®^ V2 (Kinect)	Kinect is ideal for identifying spine movement angles for game implementation, with no noticeable delay in sending data and precisely depicting the player’s movement.
RQ 2	2017 (30)	Upper limb motor coordination and function	Virtual serious games	The findings support the use of the suggested method in a longer experimental treatment to evaluate the approach’s benefits in motor function outcomes.
RQ 2	2019 (36)	Parkinson’s disease	Nintendo Wii (NW) Conventional exercises (CE)	In rehabilitating patients with Parkinson’s disease, the NW plus CE was statistically as beneficial as each intervention individually. Nevertheless, the adoption of this combined method offered a magnitude of therapeutic enefit greater than in other groups.
RQ 2	2018 (37)	Multiple sclerosis (Arm rehabilitation)	Serious games (Rehab@Home)	In a serious gaming method, VR proved viable and advantageous to arm function in people with multiple sclerosis, although the strategy’s motivating features may require additional research.
RQ 2	2017 (38)	Motor rehabilitation (Upper limb and movement/balance)	Serious games	Although serious games are more successful in enhancing upper limb motor and movement/balance functions than traditional rehabilitation, no observable variations were found in the contributions of different game aspects to effects.
RQ 2	2019 (47)	Multiple sclerosis (Unilateral arm rehabilitation)	VR-based serious games	In individuals with multiple sclerosis who have moderate to severe disabilities, an in-clinic intervention using a serious-games VR approach substantially influenced arm recovery, mostly enhancing the treated arm but also showing positive effects on the untreated arm.
RQ 2	2020 (48)	Stroke (Upper arm)	Video games, Conventional rehabilitation	In summary, no clear evidence indicates that video gaming and traditional OT resulted in the differing long-term sensorimotor recovery of the upper limb following subacute stroke. Nevertheless, when used during the first month following a stroke, video gaming outperformed traditional therapy in terms of gross grasping ability and sensorimotor recovery.
RQ 2	2015 (46)	Stroke (Upper extremity)	AMSTAR-tool	This systematic review overview offers a thorough systematic synthesis of the data regarding which primary outcomes exhibit a high degree of measurement quality and clinical utility and which may be regarded as the most appropriate for upper extremity assessment following a stroke.
RQ 3	2018 (9)	Acquired brain injury	Ship game	Patients are highly motivated to train and expedite their recovery rate.
RQ 3	2018 (15)	Traumatic brain injury (Attention deficits)	Video game tasks: Square click number tower	Training in attention that induces flow was linked to substantial improvements in attention.
RQ 3	2018 (16)	Traumatic brain injury	CogniFit Digital games designed for Sony PlayStation 3 (PS3)	No differences in improvements of the outcome measures were observed between the groups. Nevertheless, entertainment gaming elements should be considered for rehabilitative reasons.
RQ 3	2013 (23)	Brain injuries	VR equipment	Adopting VR-enhanced rehabilitation techniques has a moderate potential for producing good long-term results in patients with traumatic brain injuries with gait and balance difficulties according to the van Tulder criteria.
RQ 3	2017 (27)	Acquired brain injury	The Parrot software programme (Parrot Software, West Bloomfield, MI)	Individuals with acquired brain injury frequently have chronic and persistent residual cognitive impairments. Memory and attention are frequently the most severely damaged cognitive areas.
RQ 3	2013 (28)	Traumatic brain injury	Meta-cognitive problem-solving strategy to perform self-identified daily tasks	Remarkably, both trained and untrained objectives showed good benefits. Most effects persisted at the follow-up. Occupational therapists are recommended to use this strategy when working with adult patients with traumatic brain injury who live in the community.
RQ 3	2016 (29)	Traumatic brain injury	Attention Process Training-3 Lumosity	Individual growth curve studies revealed that participants displayed considerable progress with both therapies. Nevertheless, relatively minimal generalisation occurred. One participant showed significant improvement on one of the five probing measures.
RQ 3	2020 (21)	Neuropsychological disorders	Computerised Cognitive Training Programmes (CCTP)	Conclusively, future randomised, double-blind, controlled studies are needed to evaluate the long-term effects of CCTP in everyday activities utilising standardised outcome measures and comparing the experimental training to control training with equivalent motivation and intensity levels.
RQ 3	2019 (23)	Attention memory	Video games on measures of selective attention and visuospatial working memory (WM)	Participants increased their gaming performance throughout the training sessions. The findings of the transfer tasks suggest that game training helped both groups equally. They had better visuospatial memory and were less distracted.
RQ 3	2014 (34)	Cerebral palsy	Serious games	No conclusive evidence in the literature indicates that serious games might benefit children with cerebral palsy. Nonetheless, it should be noted that most existing treatments for children with cerebral palsy that are used on a daily basis are not supported by scientific research.
RQ 3	2017 (35)	Stroke	VR-based serious games	In contrast to the control group, the intervention group showed improved performance in memory and attention, as shown in the findings.
RQ 4	2014 (10)	Stroke	Non-invasive brain stimulators, Neuroprosthesis, VR, Wearable devices	These approaches increase motor function, rather than enhancing cognitive function or activity performance.
RQ 4	2019 (14)	Chronic hemiplegic stroke (Motor recovery)	VR training session using Xbox-Kinect	Utilising additional VR training combined with the Xbox Kinect gaming platform is a successful therapeutic strategy for enhancing motor function throughout stroke rehabilitation.
RQ 4	2016 (18)	Cerebral palsy	Xbox 360 Kinect^®^ (Microsoft)	Xbox 360 Kinect^®^ protocol has demonstrated improvements in balance and activities of daily living (ADL) among participants with cerebral palsy in a school setting.
RQ 4	2019 (23)	Chronic stroke	Xbox-Kinect	As a tool to promote whole-body movement, Xbox-Kinect games can be played by people with chronic stroke who have a range of motor abilities under the supervision of therapists.
RQ 4	2014 (26)	Impairment-based individualised rehabilitation	iPad-based software	Although both groups showed improvement, experimental participants exhibited more changes than control participants when examining only assisted or both types of sessions.
RQ 4	2014 (34)	Motor functions	Video games	Overall findings indicate that video game-based rehabilitation is at least as effective as traditional treatment. Most patients, except for some older adults, prefer video games to regular exercises. Therefore, video games should be included in the treating several pathologies.
RQ 4	2015 (39)	Neurologic disorders	Motor-cognitive dual-task training (DTT)	Although the variety of training methods and outcome measurements in the existing research made comparison of the findings across studies challenging, motor-cognitive dual-task impairments in people with neurologic diseases seem to be amenable to training. Balance, cognition, and gait may improve in people with neurologic disorders if their dual-task ability is enhanced.
RQ 4	2014 (40)	Idiopathic fallers (Gait and cognitive function)	Treadmill training (TT)	Older adults with frequent falls showed improved results in functional performance tasks, cognition, and mobility after 6 weeks of the TT plus dual task (DT) programme. Therapists may easily use DTT as a part of a training programme to lower the risk of falling.
RQ 4	2014 (45)	Stroke (Upper extremity motor function)	Computer game-based training	Because this study demonstrated increased in motor function and activity capacity, computer game-based training may be used to improve upper extremity function in the late phase following stroke.
RQ 5	2013 (11)	Stroke (Upper extremity)	VR gaming	More efficient than traditional procedures.
RQ 5	2020 (13)	Cerebral palsy	Nintendo Wii-Fit	When combined with NDT therapy, Wii Fit balance-based video games are more effective in enhancing static and performance-related balance parameters in children with mild cerebral palsy.
RQ 5	2020 (49)	Stroke	TPS system (Pressure sensor, air grip bulb)	Game-based exercises are more successful than physical training in increasing muscular strength, compliance, and motor function in patients with stroke.
RQ 5	2020 (17)	Stroke (Side brain injury)	Xbox 360 Kinect^®^	The VR game worked better for patients with right brain injury.
RQ 5	2015 (19)	Multiple sclerosis	Xbox 360 Kinect^®^	The VR system enables the optimisation of sensory information processing and integration systems, that are required to preserve the balance and PC of patients with multiple sclerosis.
RQ 5	2009 (20)	Spinal cord injury (Balance training)	Semi-immersive VR therapy	VR is an adjunctive therapy for balance rehabilitation among patients with spinal cord injury.
RQ 5	2014 (26)	Neurorehabilitation	VR	This study’s findings justify using VR as a part of a neurorehabilitation programme to maximise recovery.
RQ 5	2015 (25)	Neurorehabilitation (Multiple cognitive domains)	VR	Mobile-based VR is notably well suited to specific VR material that can be beneficial for memory rehabilitation, such as videos in 360°.
RQ 5	2014 (26)	Stroke	VR (Reh@City)	Cognitive rehabilitation with the Reh@City, an ecologically valid VR system for ADL training, has a greater impact than those of traditional techniques.
RQ 5	2012 (31)	Physical rehabilitation	Kinect, 3D cameras, Accelerometers, Balance board, Keyboard	Playing the mini-games promises genuine motivation for users; however, it should be the first rehabilitation. Sensors should give reliable measurements to determine exercise accuracy and make the games (and background analytics) clinically practical.
RQ 5	2019 (41)	Facial palsy rehabilitation	Electroencephalogram (EEG) and Electromyography (EMG) combined VR gaming system	The research on EMG and EEG demonstrated a progressive improvement in muscle production in response to impulsive and unexpected activities in the virtual world supplied by immersive VR technology.
RQ 5	2019 (50)	Parkinson’s disease, Poststroke (PS), Multiple sclerosis, Traumatic brain injury	VR-based rehabilitation	In clinical practice, VR-based therapy is feasible and successful. Considerable improvements were observed in the functional measures of gait and balance after VR-based treatments.
RQ 5	2019 (6)	Parkinson’s disease (Gait and balance)	VR-based rehabilitation	Based on the study’s findings, VR rehabilitation training does not have a similar effect as that of a traditional rehabilitation training. Furthermore, it improves balance and gait in patients with Parkinson’s disease.

## References

[1] Elaklouk AM, Zin NAM, Shapii A (2015). Investigating therapists’ intention to use serious games for acquired brain injury cognitive rehabilitation. J King Saud Univ Comput Inf Sci.

[2] Iosa M, Morone G, Fusco A, Bragoni M, Coiro P, Multari M (2012). Seven capital devices for the future of stroke rehabilitation. Stroke Res Treat.

[3] Karamians R, Proffitt R, Kline D, Gauthier LV (2020). Effectiveness of virtual reality- and gaming-based interventions for upper extremity rehabilitation poststroke: a meta-analysis. Arch Phys Med Rehabil.

[4] Merilampi S, Sirkka A, Leino M, Koivisto A, Finn E (2014). Cognitive mobile games for memory impaired older adults. J Assist Technol.

[5] Moustafa AA, Chakravarthy S, Phillips JR, Crouse JJ, Gupta A, Frank MJ (2016). Interrelations between cognitive dysfunction and motor symptoms of Parkinson’s disease: behavioral and neural studies. Rev Neurosci.

[6] Lei C, Sunzi K, Dai F, Liu X, Wang Y, Zhang B (2019). Effects of virtual reality rehabilitation training on gait and balance in patients with Parkinson’s disease: a systematic review. PLoS ONE.

[7] Park JS, Lee G, Choi JB, Hwang NK, Jung YJ (2019). Game-based hand resistance exercise versus traditional manual hand exercises for improving hand strength, motor function, and compliance in stroke patients: a multi-center randomized controlled study. NeuroRehabil.

[8] Yoshida K, Ogawa K, Mototani T, Inagaki Y, Sawamura D, Ikoma K (2018). Flow experience enhances the effectiveness of attentional training: a pilot randomized controlled trial of patients with attention deficits after traumatic brain injury. NeuroRehabil.

[9] Välimäki M, Mishina K, Kaakinen JK, Holm SK, Vahlo J, Kirjonen M (2018). Digital gaming for improving the functioning of people with traumatic brain injury: randomized clinical feasibility study. J Med Internet Res.

[10] Fernandes ABGS, Jacilda ODP, Deyvson PDB, Tania FC (2014). Comparison of the immediate effect of the training with a virtual reality game in stroke patients according side brain injury. NeuroRehabil.

[11] Gutiérrez RO, del Río FG, de la Cuerda RC, Isabel MAD, González RA, Page JCM (2013). A telerehabilitation program by virtual reality-video games improves balance and postural control in multiple sclerosis patients. NeuroRehabil.

[12] Luna-Oliva L, Ortiz-Gutiérrez RM, de La Cuerda RC, Piédrola RM, Isabel MAD, Sánchez-Camarero C (2013). Kinect Xbox 360 as a therapeutic modality for children with cerebral palsy in a school environment: a preliminary study. NeuroRehabil.

[13] Sengupta M, Gupta A, Khanna M, Krishnan UKR, Chakrabarti D (2020). Role of virtual reality in balance training in patients with spinal cord injury: a prospective comparative pre-post study. Asian Spine J.

[14] Pereira LB, Barbosa DD, Goncalves RS Development of games controlled by Kinect to spine physical therapy.

[15] Schaham NG, Zeilig G, Weingarden H, Rand D (2018). Game analysis and clinical use of the Xbox-Kinect for stroke rehabilitation. Int J Rehabil Res.

[16] Massetti T, da Silva TD, Crocetta TB, Guarnieri R, de Freitas BL, Lopes PB (2018). The clinical utility of virtual reality in neurorehabilitation: a systematic review. J Cent Nerv Syst Dis.

[17] Riva G, Mancuso V, Cavedoni S, Stramba-Badiale C (2020). Virtual reality in neurorehabilitation: a review of its effects on multiple cognitive domains. Expert Rev Med Devices.

[18] Faria AL, Andrade A, Soares L, Badia SBI (2016). Benefits of virtual reality based cognitive rehabilitation through simulated activities of daily living: a randomized controlled trial with stroke patients. J Neuroeng Rehabil.

[19] Li K, Alonso J, Chadha N, Pulido J (2015). Does generalization occur following computer-based cognitive retraining? An exploratory study. Occup Ther Health Care.

[20] Dawson DR, Gaya A, Hunt A, Levine B, Lemsky C, Polatajko HJ (2009). Using the cognitive orientation to occupational performance (CO-OP) with adults with executive dysfunction following traumatic brain injury. Can J Occup Ther.

[21] Oldrati V, Corti C, Poggi G, Borgatti R, Urgesi C, Bardoni A (2020). Effectiveness of computerized cognitive training programs (CCTP) with game-like features in children with or without neuropsychological disorders: a meta-analytic investigation. Neuropsychol Rev.

[22] Zickefoose S, Hux K, Brown J, Wulf K (2013). Let the games begin: a preliminary study using Attention Process Training-3 and Lumosity™ brain games to remediate attention deficits following traumatic brain injury. Brain Injury.

[23] Ruiz-Marquez E, Prieto A, Mayas J, Toril P, Reales JM, Ballesteros S (2019). Effects of nonaction videogames on attention and memory in young adults. Games Health J.

[24] Des Roches CA, Balachandran I, Ascenso EM, Tripodis Y, Kiran S (2015). Effectiveness of an impairment-based individualized rehabilitation program using an iPad-based software platform. Front Human Neurosci.

[25] Shute VJ, Ventura M, Ke F (2015). The power of play: the effects of Portal 2 and Lumosity on cognitive and noncognitive skills. Comp Edu.

[26] Kiran S, des Roches C, Balachandran I, Ascenso E (2014). Development of an impairment-based individualized treatment workflow using an iPad-based software platform. Semin Speech Language.

[27] Asghar I, Cang S, Yu H Software based assistive technologies for people with dementia: current achievements and future trends.

[28] Tompkins EK (2013). Brain games for cognitive improvement. J Consum Health Internet.

[29] Wentink MM, Berger MAM, de Kloet AJ, Meesters J, Band GPH, Wolterbeek R (2016). The effects of an 8-week computer-based brain training programme on cognitive functioning, QoL and self-efficacy after stroke. Neuropsychol Rehabil.

[30] Bortone I, Leonardis D, Solazzi M, Procopio C, Crecchi A, Bonfiglio L Integration of serious games and wearable haptic interfaces for neuro rehabilitation of children with movement disorders: a feasibility study.

[31] Omelina L, Jansen B, Bonnechère B, Van Sint Jan S, Cornelis J Serious games for physical rehabilitation: designing highly configurable and adaptable games.

[32] Bonnechère B, Jansen B, Omelina L, Van Sint Jan S (2016). The use of commercial video games in rehabilitation: a systematic review. Int J Rehabil Res.

[33] Sajan JE, John JA, Grace P, Sabu SS, Tharion G (2017). Wii-based interactive video games as a supplement to conventional therapy for rehabilitation of children with cerebral palsy: a pilot, randomized controlled trial. Dev Neurorehabil.

[34] Bonnechère B, Jansen B, Omelina L, Degelaen M, Wermenbol V, Rooze M (2014). Can serious games be incorporated with conventional treatment of children with cerebral palsy? A review. Res Dev Disabil.

[35] Gamito P, Oliveira J, Coelho C, Morais D, Lopes P, Pacheco J (2017). Cognitive training on stroke patients via virtual reality-based serious games. Disabil Rehabil.

[36] Santos P, Machado T, Santos L, Ribeiro N, Melo A (2019). Efficacy of the Nintendo Wii combination with conventional exercises in the rehabilitation of individuals with Parkinson’s disease: a randomized clinical trial. NeuroRehabil.

[37] Jonsdottir J, Bertoni R, Lawo M, Montesano A, Bowman T, Gabrielli S (2018). Serious games for arm rehabilitation of persons with multiple sclerosis: a randomized controlled pilot study. Multiple Sclerosis Rel Disord.

[38] Tǎut D, Pintea S, Roovers JPWR, Mañanas MA, Bǎban A (2017). Play seriously: effectiveness of serious games and their features in motor rehabilitation: a meta-analysis. NeuroRehabil.

[39] Fritz NE, Cheek FM, Nichols-Larsen DS (2015). Motor-cognitive dual-task training in persons with neurologic disorders: a systematic review. J Neurol Physic Ther.

[40] Dorfman M, Herman T, Brozgol M, Shema S, Weiss A, Hausdorff JM (2014). Dual-task training on a treadmill to improve gait and cognitive function in elderly idiopathic fallers. J Neurol Physic Ther.

[41] Qidwai U, Ajimsha MS, Shakir M (2019). The role of EEG and EMG combined virtual reality gaming system in facial palsy rehabilitation: a case report. J Bodyw Move Ther.

[42] Piau A, Wild K, Mattek N, Kaye J (2019). Current state of digital biomarker technologies for real-life, home-based monitoring of cognitive function for mild cognitive impairment to mild Alzheimer disease and implications for clinical care: systematic review. J Med Internet Res.

[43] Ge S, Zhu Z, Wu B, McConnell ES (2018). Technology-based cognitive training and rehabilitation interventions for individuals with mild cognitive impairment: a systematic review. BMC Geriatr.

[44] Jirayucharoensak S, Israsena P, Pan-ngum S, Hemrungrojn S, Maes M (2019). A game-based neurofeedback training system to enhance cognitive performance in healthy elderly subjects and in patients with amnestic mild cognitive impairment. Clin Intervent Aging.

[45] Slijper A, Svensson KE, Backlund P, Engström H, Sunnerhagen KS (2014). Computer game-based upper extremity training in the home environment in stroke persons: a single subject design. J Neuroeng Rehabil.

[46] Murphy MA, Resteghini C, Feys P, Lamers I (2015). An overview of systematic reviews on upper extremity outcome measures after stroke. BMC Neurol.

[47] Jonsdottir J, Perini G, Ascolese A, Bowman T, Montesano A, Lawo M (2019). Unilateral arm rehabilitation for persons with multiple sclerosis using serious games in a virtual reality approach: bilateral treatment effect?. Multiple Sclerosis Rel Disord.

[48] Laffont I, Froger J, Jourdan C, Bakhti K, van Dokkum LEH, Gouaich A (2020). Rehabilitation of the upper arm early after stroke: Video games versus conventional rehabilitation: a randomized controlled trial. Ann Phys Rehabil Med.

[49] Aulisio MC, Han DY, Glueck AC (2020). Virtual reality gaming as a neurorehabilitation tool for brain injuries in adults: a systematic review. Brain Injury.

[50] Cano PD, Sharon H, Inzelberg R, Ziv-Ner Y, Zeilig G, Plotnik M (2019). Advanced virtual reality-based rehabilitation of balance and gait in clinical practice. Ther Adv Chronic Dis.

